# The Protective Effect of Polyherbal Formulation, Harak Formula, on UVA-Induced Photoaging of Human Dermal Fibroblasts and Mouse Skin *via* Promoting Nrf2-Regulated Antioxidant Defense

**DOI:** 10.3389/fphar.2021.649820

**Published:** 2021-04-12

**Authors:** Jinapath Lohakul, Anyamanee Chaiprasongsuk, Saowanee Jeayeng, Malinee Saelim, Phetthinee Muanjumpon, Saowalak Thanachaiphiwat, Pinpat Tripatara, Kittipong Soontrapa, Natchagorn Lumlerdkij, Pravit Akarasereenont, Uraiwan Panich

**Affiliations:** ^1^ Department of Pharmacology, Faculty of Medicine Siriraj Hospital, Mahidol University, Bangkok, Thailand; ^2^ Faculty of Medicine and Public Health, HRH Princess Chulabhorn College of Medicine Science, Chulabhorn Royal Academy, Bangkok, Thailand; ^3^ Center of Applied Thai Traditional Medicine, Faculty of Medicine Siriraj Hospital, Mahidol University, Bangkok, Thailand

**Keywords:** polyherbal formulation, photoaging, nuclear factor E2-related factor 2 (Nrf2), ultraviolet a, fibroblasts

## Abstract

Polyherbal formulation combining multiple herbs is suggested to achieve enhanced therapeutic effects and reduce toxicity. Harak herbal formula (HRF) extracts were proposed to regulate skin responses to UVR through their ability to suppress UVA-induced matrix metalloproteinase-1 (MMP-1) and pigmentation via promoting antioxidant defenses in *in vitro* models. Therefore, natural products targeting Nrf2 (nuclear factor erythroid 2-related factor 2)-regulated antioxidant response might represent promising anti-photoaging candidates. Hesperetin (HSP) was suggested as a putative bioactive compound of the HRF, as previously shown by its chemical profiling using the liquid chromatography coupled with quadrupole time-of-flight mass spectrometry (LC-QTOF-MS). In this study, we explored the anti-photoaging effects of HRF extracts and HSP on normal human dermal fibroblasts (NHDFs) and mouse skin exposed to UVA irradiation. Pretreatment of NHDFs with HRF extracts and HSP protected against UVA (8 J/cm^2^)-mediated cytotoxicity and reactive oxygen species (ROS) formation. The HRF and HSP pretreatment also attenuated the UVA-induced MMP-1 activity and collagen depletion concomitant with an upregulation of Nrf2 activity and its downstream genes (GST and NQO-1). Moreover, our findings provided the *in vivo* relevance to the *in vitro* anti-photoaging effects of HRF as topical application of the extracts (10, 30 and 100 mg/cm^2^) and HSP (0.3, 1, and 3 mg/cm^2^) 1 h before UVA exposure 3 times per week for 2 weeks (a total dose of 60 J/cm^2^) mitigated MMP-1 upregulation, collagen loss in correlation with enhanced Nrf2 nuclear accumulation and its target protein GST and NQO-1 as well as reduced 8-hydroxy-2′-deoxyguanosine (8-OHdG) in irradiated mouse skin. Thus, our findings revealed that HRF extracts and HSP attenuated UVA-induced photoaging via upregulating Nrf2, together with their abilities to reduce ROS formation and oxidative damage. Our study concluded that the HRF and its bioactive ingredient HSP may represent potential candidates for preventing UVA-induced photoaging via restoration of redox balance.

**Figure:**
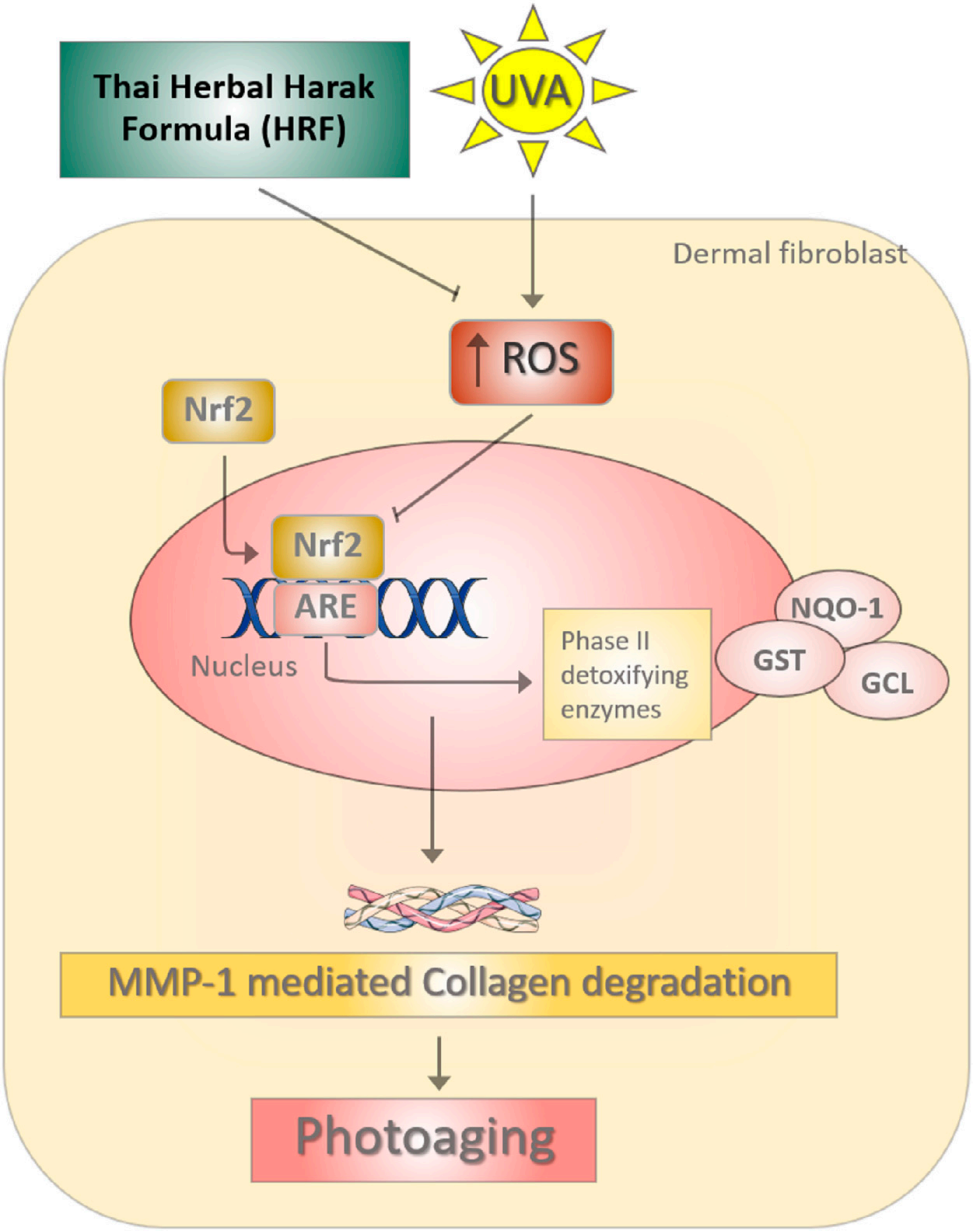
**GRAPHICAL ABSTRACT | **The photoprotective role of Thai Herbal Harak formula (HRF) and its possible active compound hesperetin (HSP) in UVA-induced photoaging through redox-dependent regulation of Nrf2. During the UVA irradiation, the imbalance of reactive oxygen species (ROS) causes the down-regulation of Nrf2-regulated antioxidant defense including GST and NQO-1, leading to the promotion of the MMP-1 responsible for collagen degradation. HRF could represent a promising strategy for delaying the process of photoaging via the inhibition of MMP-1.

## Introduction

The concept of polyherbal formulation or herb-herb combinations has been found in the Ayurvedic system of medicine and other traditional systems of herbal medicine such as Unani and Chinese medicine practice. While polyherbal therapy has been widely practiced for thousands of years, there is limited scientific evidence to establish their pharmacological effects and safety. Therefore, reliable evidence is required to support the proposed health claims. Polyherbal formulation combining multiple medicinal herbs is suggested to provide an enhanced therapeutic effect and reduce toxicity (Parasuraman et al., 2014). The demand for topical herbal remedies and cosmetics is growing worldwide and polyherbal therapy for skin disorders has also attracted considerable attention (Tabassum and Hamdani, 2014; Talekar et al., 2017). A Thai traditional polyherbal formulation Harak herbal formula (HRF) or Benchalokawichian has been traditionally used not only to treat fever but also to improve skin ailments as well as promote youthful skin (Juckmeta et al., 2014). The HRF is composed of roots of five plants in equal amounts: *Ficus racemosa L. (Moraceae), Capparis micracantha DC. (Capparidaceae), Clerodendrum petasites (Lour.) S. Moore (Lamiaceae), Harrisonia perforata (Blanco) Merr. (Rutaceae), and Tiliacora triandra (Colebr.) Diels (Menispermaceae)*. Various chemical constituents identified in the extracts of HRF and their components include alkaloids, coumarins, flavonoids, phenolic compounds, tannins, and triterpenoids (Khan and Sultana, 2005; Mahidol et al., 2009). As demonstrated by the chemical profiling of HRF and all five components (the roots of five plants used in preparation of the HRF) using the liquid chromatography coupled with quadrupole time-of-flight mass spectrometry (LC-QTOF-MS), hesperetin (HSP) (or the (2S)-5,7-dihydroxy-2-(3-hydroxy-4-methoxyphenyl)-2,3-dihydrochromen-4-one) was also suggested as a putative bioactive constituent of the HRF (Suebnooch et al., 2018). We and others have reported the beneficial roles of the HRF and their components in protecting against skin disorders possibly due to several pharmacological actions *in vitro* and *in vivo* including anti-acne (Jantarat et al., 2018), anti-allergic (Juckmeta et al., 2014), anti-inflammatory (Juckmeta et al., 2019), antioxidant and anti-collagenase (Pluemsamran et al., 2013), depigmenting (Onkoksoong et al., 2018), and wound healing properties (Mehtaa et al., 2012).

Regarding the continuous increase in the aging population worldwide, research of age-related redox imbalance driving pathological conditions including skin photoaging is needed to develop pharmacological strategies to slow the aging process or improve the quality of life. Chronic UVA and UVB irradiation is considered as the major environmental factor causing cutaneous photodamage associated with premature aging or photoaging and photocarcinogenesis. UVA irradiation (320–400 nm) can cause premature aging of the skin through triggering stimulation of matrix metalloproteinase-1 (MMP-1) responsible for collagen degradation, a hallmark of photoaged skin (Nakyai et al., 2017). Moreover, UVB (280–320 nm) plays a role in cutaneous photodamage through apoptosis and DNA damage of skin cells including melanocytes (Jeayeng et al., 2017). Previous studies including our reports have suggested that oxidative stress, an imbalance between reactive oxygen species (ROS) production and the antioxidant defenses, plays a crucial role in the upregulation of MMP-1 in keratinocyte HaCaT cells (Pluemsamran et al., 2012; Kim et al., 2015), and mouse skin following UVA exposure.

Nuclear factor E2-related factor 2 (Nrf2) is a master transcription factor regulating several phase II detoxification genes, such as glutathione S-transferase (GST) and NAD(P)H:quinone oxidoreductase-1 (NQO-1), involved in cellular defenses against oxidative stress and environmental insults including UVR (Gegotek and Skrzydlewska, 2015). Upregulation of Nrf2 signaling was also observed in previous studies to mitigate the effects of UVA on oxidative, inflammatory and apoptotic responses of the skin cells including keratinocytes and melanocytes (Chaiprasongsuk et al., 2016) and dermal fibroblasts (Hirota et al., 2005; Lu et al., 2020) We thus proposed that regulation of nuclear factor E2-related factor 2 (Nrf2), the crucial regulator of antioxidant and cytoprotective genes, to improve the redox balance could represent a promising pharmacological target for the development of herbal medicines as effective anti-photoaging strategies. Evidence-based medicine of the herbal candidates and their bioactive compounds is required to support further development of the HRF as promising topical anti-photoaging agents. In this study, we thus investigated the protective effects of HRF and its putative bioactive compound HSP on UVA-induced photoaging of normal human dermal fibroblasts (NHDFs) and mouse skin via upregulating Nrf2-regulated antioxidant defense.

## Materials and Methods

### Preparation of HRF Extracts

HRF powder was produced by Ayurved Siriraj Manufacturing Unit of Herbal Medicines and Products, Bangkok, Thailand (a GMP-certified manufacturer). Briefly, all ingredients were washed, dried, ground, and mixed to obtain a uniform batch of HRF powder. Then the powder was inspected for its quality, such as loss on drying, particle size distribution, powder flow, and density, to ensure that it conformed with the specification. HRF extract was prepared by mixing 50 g of HRF powder with 500 ml of 80% ethanol for 30 min, followed by 30 min ultrasonication. After filtration through a Whatman filter paper, the solvent was removed using a rotary evaporator and a freeze dryer. The dry extract was kept at room temperature until used.

We previously performed the qualitative analysis of the HRF by LC-QTOF-MS in both positive and negative electrospray ionization (ESI) modes to investigate chemical profiles and structures. The chemical structure of 5,7-Dihydroxy-2-(3-hydroxy4-methoxyphenyl)-2,3-dihydro-4H-chromen-4-one or “hesperetin (HSP)” was identified in positive ESI mode at the retention time of 4.9435 and the negative ESI mode at the retention time of 4.9827 and was expressed in all HRF’s five herb components (Suebnooch et al., 2018).

### Cell Culture and Treatment

Full details are supplied in Supplementary Material. Normal human dermal fibroblasts (NHDFs) cells (CC-2511: Lonza, Basel, Switzerland) were grown in a high glucose (4.5 g/l) of the Dulbecco's Modified Eagle's Medium (DMEM) supplemented with 10% fetal bovine serum (FBS) and 1% penicillin (100 units/ml)/streptomycin (P/S) (100 μg/ml) at 37°C in a humidified air of 5% CO_2_ (P_CO2_ = 40 Torr). The NHDFs were then assigned to experimental treatments after washing by phosphate-buffered saline (PBS). The HRF and HSP (CAS 69097: Sigma–Aldrich), the HRF’s putative bioactive compound, were dissolved in 80% ethanol. Therefore, the vehicle-sham or the vehicle control group was the NHDFs treated with 80% ethanol (the final concentration not exceeding 0.08%). The treatment groups were therefore compared with the vehicle-sham group with or without UVA exposure. In the *in vitro* study, three-different concentrations: HRF at the concentration of 7.5, 15, and 30 μg/ml and HSP at the concentration of 2.5, 5, and 10 μg/ml, were obtained to perform in the experiments for determination of cytotoxicity, ROS formation, photoaging parameters, and Nrf2-regulated antioxidant response.

### UVA Irradiation

After the compound treatments, a thin layer of PBS (100 μl/cm^2^) was added to the cells then the cells were irradiated with UVA. The UV-meter (Hand-held UV-meter, Honle UV technology, Germany) equipped with a UVA sensor (330–400 nm) was obtained to evaluate the constant intensity of UVA ray, 1 W/cm^2^ at a 21 cm distancing from the xenon arc UVA lamp (Dermalight ultrA1; Hoenle, Martinsried, Germany). The irradiation of UVA (8 J/cm^2^) was performed after the cell treatment with each compound. Irradiated cells were immediately incubated with serum-free medium, which were then harvested at different time points; 12 h post-irradiation for cytotoxicity, 1 h post-irradiation for ROS formation, Nrf2 nuclear translocation (Chaiprasongsuk et al., 2016), 24 h post-irradiation for MMP-1 activity, MMP-1 and collagen type I expression, 12 and 2 h post-irradiation for the expressions of MMP-1 and Nrf2 target genes (Chaiprasongsuk et al., 2017), respectively.

### Determination of ROS Production by Flow Cytometry

At 1 h post-irradiation, treated cells were incubated in PBS with 5 μm non-fluorescent dichlorofluorescein (H_2_DCFDA) at 37°C for 30 min. Permeated H_2_DCFDA was deacetylated by intracellular esterases to non-fluorescent product (H_2_DCF). Stained cells were immediately analyzed by flow cytometry (FACS-Calibur) as previously described (Chaiprasongsuk et al., 2017). Data were expressed as a percentage of control (100%, sham treatment in non-irradiated cells).

### Determination of Cytotoxicity by Flow Cytometry

The cytotoxicity was evaluated by using the Propidium iodide (PI) according to the manufacturer’s instructions (BD Biosciences, United States). After the pre-treatment of the cells with compounds, the cells were irradiated with UVA 10 J/cm^2^ and further incubated in a serum-free medium for 12 h. The cells were then washed and stained with PI dye before performing the flow cytometry detection as described in our previous report (Chaiprasongsuk et al., 2017). Data were subsequently analyzed by FlowJo Software ver. 10 and expressed as a percentage of control (100%, sham treatment in non-irradiated cells).

### Detection of MMP-1 Activity by Collagen Zymography

The conditioned media collected at 24 h after UVA exposure was used to detect MMP-1 activity using collagen zymography as previously described (Flament et al., 2013). Culture supernatant was subjected to electrophoresis on 10% polyacrylamide gels containing 1% collagen substrate. Following electrophoresis, the gel was washed twice with 2.5% Triton X-100 to remove SDS and allow MMP-1 to renature and was incubated in the developing buffer overnight at 37°C. The gel was then stained with 0.006% Coomassie brilliant blue G-250 and destained using a destaining solution as previously described in Chaiprasongsuk et al., 2017. Determination of MMP-1 activity observed as colorless (unstained) bands was performed using a CAMAG TLC scanner (Muttenz, Switzerland) and analyzed with the ImageMaster software (Hoefer Pharmacia Biotech). Data were expressed as a percentage of control (100%, sham treatment in non-irradiated cells).

### The mRNA Quantitation Using a Real-Time RT-PCR

The expressions of MMP-1 gene and Nrf2 target genes (GST and NQO-1) were determined at 12 and 2 h post-irradiation, respectively. Total RNA was isolated using the Illustra RNAspin Mini RNA Isolation Kit (GE Healthcare, United Kingdom) and the cDNA was then synthesized from total RNA using Improm-II reverse transcriptase (Promega, Medison, United States) following the manufacturer’s instructions. Primers for PCR were designed based on strict criteria using the Primer Express version 3.0 software (Applied Biosystems, United States) as listed in Table 1. The amplification of a single product was verified using the melt curve analysis and the GAPDH gene was used for the normalization of target gene expression data. The mean Ct from mRNA expression in cDNA was compared with the mean Ct from GAPDH determinations from the same cDNA samples. The procedure and analysis were described in our previous report (Chaiprasongsuk et al., 2017).

**TABLE 1:** Sequences (in 5–3′ direction) of primers used in this study.

Primer	Sequences	Product size (I et al.)	GeneBank
MMP-1 (sense)	5′-TGT​GGA​CCA​TGC​CAT​TGA​GA-3′	67	NM_002421.4
MMP-1 (antisense)	5′-CTT​GGT​GAA​TGT​CAG​AGG​TGT​GA-3′		
NQO-1 (sense)	5′-GTT​TGG​AGT​CCC​TGC​CAT​TCT-3′	163	NM_000903.3
NQO-1 (antisense)	5′-CCC​TTG​CAG​AGA​GTA​CAT​GGA​G-3′		
GST (sense)	5′-GAC​GGA​GAC​CTC​ACC​CTG​TA-3′	232	NM_000852.3
GST (antisense)	5′-ACA​GCA​GGG​TCT​CAA​AAG​GC-3′		
GAPDH (sense)	5′-GGT​GAA​GGT​CGG​AGT​CAA​CG-3′	197	NM_002046.7
GAPDH (antisense)	5′-TGA​CAA​GCT​TCC​CGT​TCT​CAG-3′		

### Western Blot Analysis of Nrf2 Nuclear Localization and the Collagen Type I Protein Expression

Treated cells were harvested at 1 h post-irradiation for the Nrf2 nuclear translocation, while, they were harvested at 24 h post-irradiation for the collagen type I protein expression. Cytosolic and nuclear extracts were carried out to detect nuclear localization of Nrf2 (sc-722; Santa Cruz Biotechnology, Santa Cruz, CA) (1:2500), while whole-cell extracts were carried out to detect the collagen type I protein expression (C-18) Ab (sc-8784; Santa Cruz Biotechnology, Santa Cruz, CA) (1:2500). For the nuclear extraction, cytosolic and nuclear extraction were prepared using a commercial kit according to the manufacturer's instructions (Sigma). The HRP-conjugated secondary antibodies (ab6789 for anti-mouse and ab6721 for anti-rabbit HRP labeled secondary antibody; Abcam, Cambridge, MA, United States) (1:2000) were used for the immunochemical detection. The immunoreactivity was then detected by the chemical reaction with the Bio-Rad Clarity western ECL (Bio-Rad). Protein bands were visualized using an ImageQuant LAS 4000 digital imaging system (GE Healthcare, United Kingdom) and the integrated optical density of the bands was analyzed by the ImageJ software version 1.45 s (National institutes of health, United States). The protein expressions were normalized to the expression of loading controls; *β*-actin (ab7291; Abcam, Cambridge, MA, United States) (1:5,000) for whole cell proteins or cytosol Nrf2 and collagen, and TATA binding protein (TBP) (ab818; Abcam, Cambridge, MA, United States) (1:2,500) for nuclear Nrf2. Data were expressed as a percentage of control (100%, sham treatment in non-irradiated cells).

### Animal Model and the Treatment

All animal experiments were reviewed and approved by the Siriraj Animal Care and Use Committee (SiACUC), SiACUP 023/2557. BALB/c mice were obtained from the National Laboratory Animal Center, Mahidol University and were housed under standard housing conditions with light: dark cycle at 12:12 h. BALB/c mice (4–5 weeks of age with an average body weight of 20 g) were anesthetized by intraperitoneal injection of a ketamine (80 mg/kg)/xylazine (10 mg/kg) cocktail. The dorsal surfaces were then shaved on using an electric hair clipper (Remington, PG-180). The scarified area was cleansed with a cotton-tipped applicator saturated with sterile normal saline solution. The three-different concentrations of each treatment, HRF at the concentrations of 10, 30 and 100 mg/cm^2^, HSP at the concentrations of 0.3, 1, and 3 mg/cm^2^ and sulforaphane (SFN) 0.1 mg/cm^2^ were obtained to perform in all biomarkers of the *in vivo* study. HRF, HSP, and SFN powders were dissolved in 20 μL of ethanol: acetone (1:1, v*:*v) and topically administered to each 1-cm^2^ site in the center area of the shaved dorsal skin 1 h prior to each UVA exposure. The mice were exposed to UVA irradiation for 10 min to achieve a single dose of 10 J/cm^2^, 3 times a week for 2 weeks (60 J/cm^2^ in total). The dorsal skin flaps were then removed at different time points. Skin thickness was assessed by hematoxylin and eosin (H&E) and immunofluorescence (IF) staining. Photoaging markers MMP-1 and collagen, oxidative DNA damage (8-OHdG), as well as Nrf2 nuclear accumulation and the protein levels of its downstream target genes GST and NQO-1, were assessed by IF. The timeline of treatment, the UVA dose selection, procedures of IF staining protocol and all analysis of the protein expression data were described below following previous reports (Shimada et al., 2011; Chaiprasongsuk et al., 2017).

### Hematoxylin and Eosin (H&E) Analyses of Skin Thickness

Frozen tissues were sectioned by using Cryostat (Thermo scientific, United States) for 8 μm per 1 section. Cryo-cut tissue sections were fixed in ice-cold acetone and air-dried for 30 min at room temperature. H&E staining was performed for histological evaluation of skin thickness as previously described in Chaiprasongsuk et al., 2017. Briefly, tissue sections were washed in distilled water for 2 min, incubated with hematoxylin for 4 min, and then washed in distilled water for 10 min. The slides were then incubated with eosin for 1 min and 95% alcohol for 1 min. The slides were dehydrated with 95% alcohol, 2 changes of absolute alcohol and acetone as well as 3 changes of xylene. An inverted fluorescent microscope equipped with a Nikon Intensilight was used for the imaging of H&E staining which was quantified using ImageJ software (Gawronska-Kozak et al., 2016; Chaiprasongsuk et al., 2017).

### Immunofluorescence Analysis of Nrf2 Nuclear Translocation and Its Target Proteins (GST, and NQO-1), Oxidative DNA Damage, MMP-1, Collagen

Dorsal skin tissue samples were collected at various time points following the final UVA irradiation; 1, and 6 h post-irradiation for Nrf2 and its target proteins, respectively; 1 h post-irradiation for oxidative DNA damage; 24 h post-irradiation for MMP-1 and collagen. Tissue sections were washed with PBS for 5 min/time (3 times) and blocked with phosphate-buffered saline (PBS) containing 2% BSA for 30 min. After removing excess blocking buffer, the slides were incubated with Nrf2 Ab (ab31163; Abcam, Cambridge, MA, United States), GST Ab (sc-459; Santa Cruz Biotechnology, Santa Cruz, CA), NQO1 Ab (ab34173; Abcam, Cambridge, MA, United States) (1:50), 8-OHdG [N45.1] Ab (ab48508; Abcam, Cambridge, MA, United States) (1:50), MMP-1 Ab (ab137332; Abcam, Cambridge, MA, United States) (1:50), collagen I (C-18) Ab (sc-8784; Santa Cruz Biotechnology, Santa Cruz, CA) (1:50) for 1 h. The slides were then washed for 5 min/time (3 times) with a PBS solution and incubated for 1 h at room temperature with FITC-conjugated the secondary Ab (green) and with DAPI (blue) to counterstain the nuclei for detection of nuclear Nrf2, the secondary Ab Alexa Fluor 488 goat anti-rabbit (Abcam) for detection of MMP-1 and collagen levels. An inverted fluorescent microscope equipped with a Nikon Intensilight was used for the imaging of IF stainings (20X) which were quantified using ImageJ software.

### Data Analysis

ImageJ software (NIH, Rockville, MD, United States was used to quantify the immunoblot intensity, thickness, and the IF intensity of the protein expressions. For all analysis of protein expression data, the corrected total cryosection fluorescence (CTCF) was calculated using the following equation: CTCF = integrated density–(area of each region of interest (ROI) × mean fluorescence of background readings) and the data were presented as percentage of control. Quantitative fluorescence analysis from ImageJ was then imported into Microsoft Excel to create histograms for further analysis. All analysis of protein expression data was presented as a percentage of control. The Nrf2 nuclear localization was analyzed based on the ratio of fluorescence intensity in the nuclear (indicated by the DAPI staining) to cytoplasmic intensity of Nrf2 (N/C ratio). Background subtraction was done to correct the intensity from each compartment (Noursadeghi et al., 2008; Chaiprasongsuk et al., 2017).

### Statistical Analysis

Means ± standard deviation from at least three biological replicates (*n* ≥ 3) were reported as a scientific value for both *in vitro* and *in vivo* studies. The significance analysis was evaluated by independent *t* test (Student’s; 2 populations) or one-way analysis of variance followed by Tukey or Dunnett tests, where appropriate, using Prism (GraphPad Software Inc. San Diego, CA) (the non-irradiated controls or individual treatment groups vs. the UVA-irradiated groups). A one-way analysis of variance followed by the appropriate post hoc analysis was used to determine individual group differences of the UVA-treated sham group vs. the groups treated with compounds and UVA exposure. The statistical significance was considered at a value of *p* < 0.05 and the significance level was defined in the figure legends.

## Results

### HRF and HSP Protected Against UVA-Induced Cytotoxicity and ROS Formation in NHDFs

Firstly, we investigated the control experiments using HRF extracts and HSP, a potential bioactive compound of the test extracts. At the highest concentration used in the *in vitro* part of our study (30 μg/ml and 10 μg/ml), each compound alone did not cause any significant cytotoxicity (Supplementary Figures S1A,B) or ROS formation (Supplementary Figures 1C,D). Preliminary results indicated UVA exposure of NHDFs led to dose-dependent ROS formation and cellular damage (data not shown), thus a UVA exposure of 8 J/cm^2^ was used for further studies. A previous study similarly reported the UVA at dose 8 J/cm^2^ can damage the skin cells through the aging process by upregulating the MMPs expression.

**FIGURE 1:**
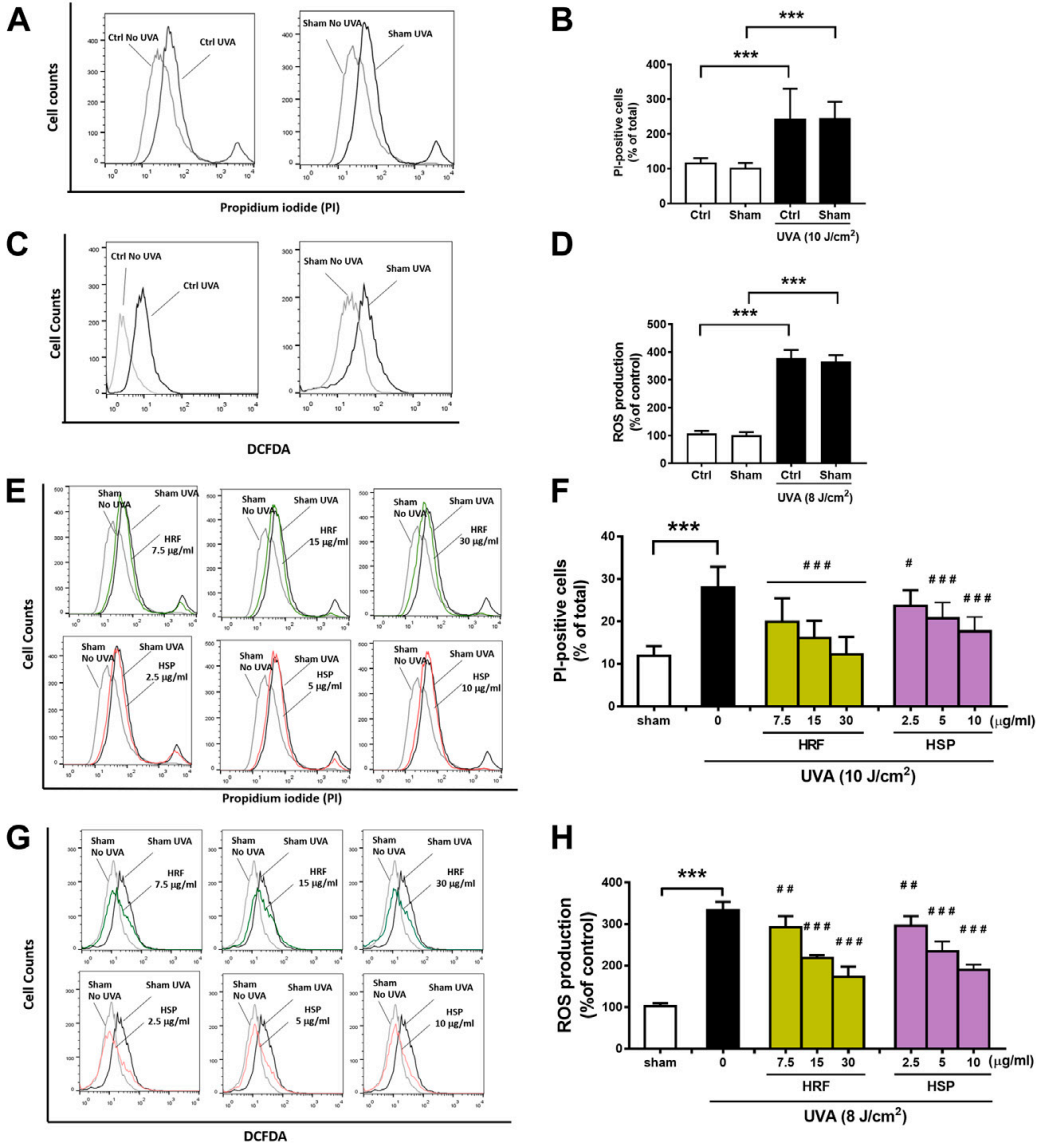
The protective effects of HRF and its active ingredient HSP on UVA-induced cytotoxicity and oxidative stress in NHDFs. HRF at dose 7.5, 15, and 30 μg/ml and HSP at dose 2.5, 5, and 10 μg/ml as well as the ethanol treatment and no compound treatment (0), were individually treated in NHDFs prior to UVA (10 J/cm^2^) irradiation for cytotoxicity assay. Treated cells were harvested at 12 h post-irradiation to perform the flow cytometry of fluorescent staining, propidium iodide (PI), for the PI-positive cells (% of total). The flow histogram **(A)** and summary graph analysis **(B)** of ethanol sham treatment and compare to UVA irradiated cells and the histogram **(E)** and summary graph analysis of treatment groups **(F)**. The HRF and HSP were individually treated in NHDFs prior to UVA (8 J/cm^2^) irradiation. Treated cells were harvested at 1 h post-irradiation to perform the flow cytometry of fluorescent staining, DCFDA dye, for the evaluation of the ROS production (% of control). The flow histogram **(C)** and summary graph analysis **(D)** of ethanol sham treatment and compare to UVA irradiated cells and the histogram were expressed as mean ± SD, *n* = 4. ***p < 0.001 vs. non-irradiated sham group by Student’s *t*-test. *#p* < 0.05; ##*p* < 0.01; ###*p* < 0.001 vs. the sham-irradiated group by one-way ANOVA Dunnett's test.

Evaluation of the cytotoxicity using PI staining and cellular ROS production using DCFDA, two standard parameters of cellular damage, was performed to investigate the effects of UVA exposure on NHDFs. The significant (*p* < 0.001) inductions of cytotoxicity (Figures 1A,B) and cellular ROS production (Figures 1C,D) were observed in the NHDFs following the UVA irradiation at doses of 10 and 8 J/cm^2^, respectively, whereas vehicle control using sham treatment of cells had no significant effect on UVA-induced cytotoxicity or ROS formation. However, treatment of NHDFs with the HRF extracts and HSP significantly inhibited UVA-induced cytotoxicity (Figures 1E,F) and cellular ROS generation (Figures 1G,H) in a concentration-dependent manner.

### HRF and HSP Protected Against UVA-Induced Photoaging of NHDFs Through Modulation of MMP-1 and Collagen

**FIGURE 2:**
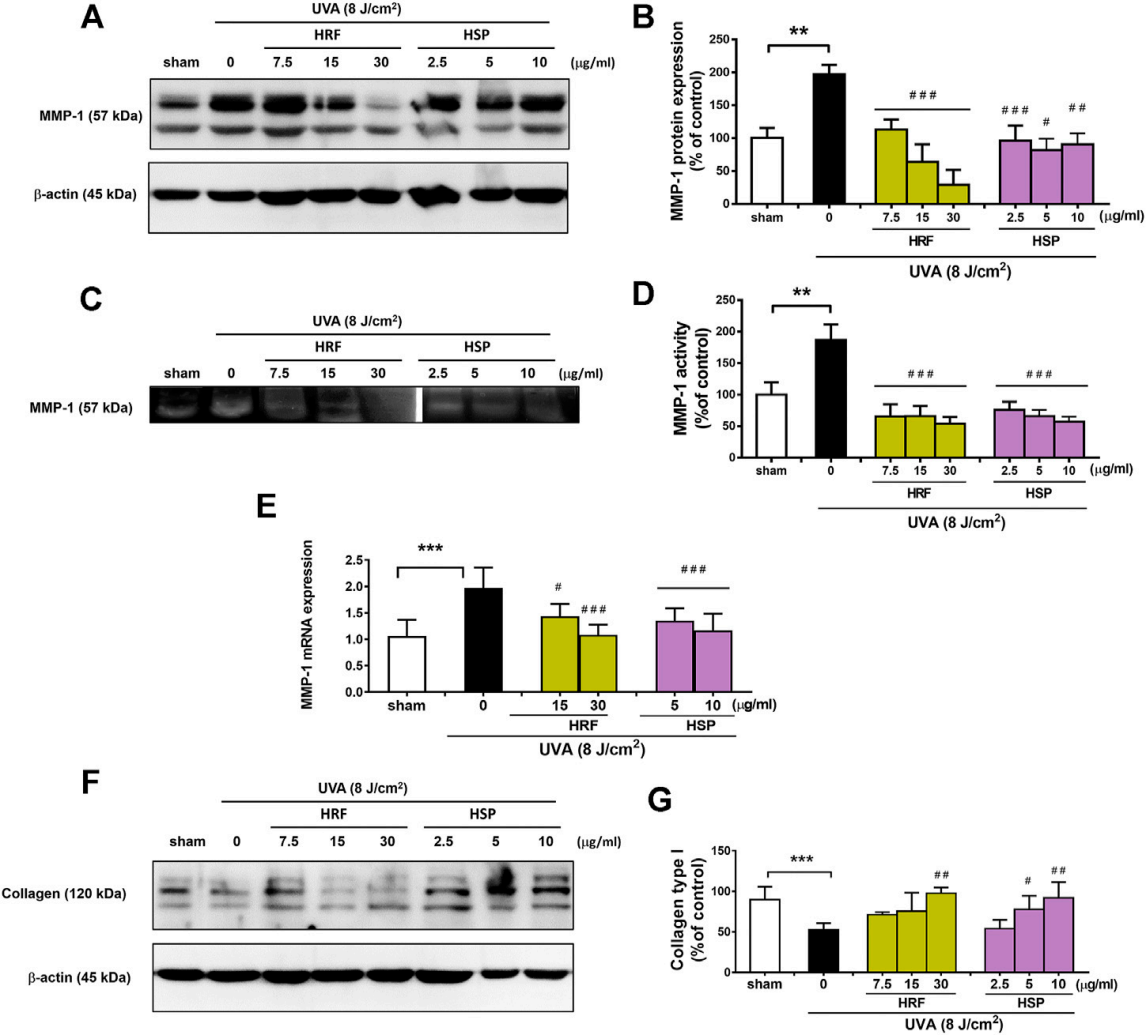
The protective effects of HRF and its active ingredient on the photodamaged skin via the alteration of MMP-1 and collagen levels. NHDFs were treated with compounds as previously described and harvested at 24 h following UVA (8 J/cm^2^) exposure for the western blot analysis of the MMP-1 protein expression with the indicated molecular weight (57 kDa), MMP-1 enzyme activity by zymography, mRNA levels of MMP-1 and western blot analysis of collagen type 1 (% of control). The representative blot pictures of **(A)** and summary graph analysis of MMP-1 protein expression **(B)**. The collagen zymography **(C)** and summary graph analysis of MMP-1 enzyme activity **(D)** and the mRNA levels of MMP-1 **(E)**. The collagen type I with the indicated molecular weight (120 kDa) **(F)** and summary graph analysis **(G)** in each compound treatment as compared to ethanol treatment. **p < 0.01 and ***p < 0.001 vs. non-irradiated sham group by Student’s *t*-test. *#p* < 0.05; ##*p* < 0.01; ###*p* < 0.001 vs. the sham-irradiated group by one-way ANOVA Dunnett's test.

A hallmark of photoaged skin is characterized by the destruction of skin collagen type I through induction of MMP-1 (or collagenase-1), one of the main enzymes responsible for the extracellular matrix (ECM) breakdown. We then investigated the two parameters, MMP-1 and collagen, of photoaging to explore the effects of HRF and HSP on these parameters. Our data showed that vehicle control using sham treatment of NHDFs had no significant effect on MMP-1 protein expression (Supplementary Figures S2A,B), MMP-1 enzyme activity (Supplementary Figures S2C,D), and collagen type 1 protein (Supplementary Figures S2E,F in UVA (8 J/cm^2^)-irradiated cells. At 24 h post-UVA irradiation, MMP-1 protein expression and enzyme activity were examined by western blotting (Figures 2A,B) and collagen zymography (Figures 2C,D), respectively, and MMP-1 mRNA levels were assessed by real-time RT-PCR at 12 h post-irradiation (Figure 2E). The UVA irradiation-induced MMP-1 protein expression (Figures 2A,B), enzyme activity (Figures 2C,D), and mRNA expression (Figure 2E) were significantly decreased by HRF and HSP. In addition, the treatment of cells with HRF and HSP significantly mitigated UVA-mediated reduction of collagen (30 μg/ml of HRF and 5–10 μg/ml of HSP) (Figures 2F,G).

### HRF and HSP Suppressed UVA-Induced Downregulation of Nrf2 Activity and Its Downstream Signaling

**FIGURE 3:**
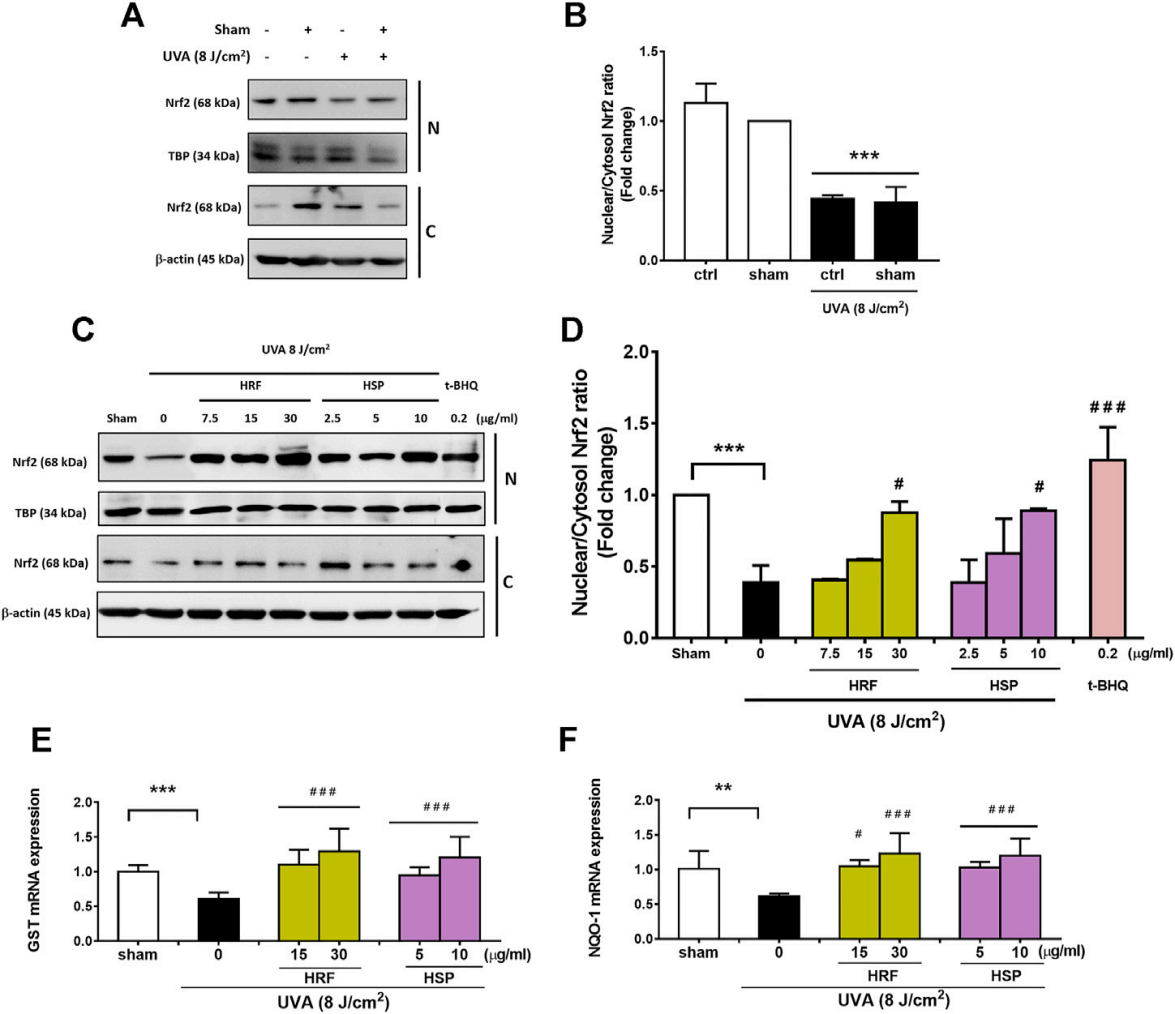
The photoprotective role of HRF and its active ingredient HSP involved the Nrf2-regulated redox balance. Following 1 h UVA (8 J/cm^2^) irradiation, NHDFs were harvested to perform the western blot analysis of Nrf2 nuclear translocation (the nuclear-to-cytosolic Nrf2 ratio) and its downstream antioxidant genes (% of control). The representative blot pictures **(A)** and the summary graph analysis of the UVA irradiated cells compared to unirradiated control (ctrl) cells and ethanol treatment in irradiated cells **(B)**. The representative blot pictures of each treatment group **(C)** and the summary graph analysis of the treatment group **(D)**. The summary graph analysis of its downstream signaling including GST **(E)**, and NQO-1 genes **(F)** in HRF or HSP treatment as compared to sham treatment were expressed as mean ± SD, *n* = 4. **p < 0.01; ***p < 0.001 vs. non-irradiated sham group by Student’s *t*-test. *#p* < 0.05; ###*p* < 0.001 vs. the sham-irradiated group by one-way ANOVA Dunnett's test.

Nrf2 is suggested to be a transcription regulator of the antioxidant defense system, contributing to the effective redox balance. The mRNA levels of Nrf2 transcriptional targets including GST and NQO-1 were then determined to study the activity of Nrf2. We thus investigated the effects of HRF and HSP in the modulation of the Nrf2-regulated cytoprotective genes including GST and NQO-1. Our findings revealed that UVA significantly reduced nuclear and cytoplasmic Nrf2 levels (Figure 3A) and nuclear: cytoplasm Nrf2 ratio (Figure 3B) at 1 h post-irradiation. Conversely, treatment of NHDFs with HRF and HSP (30 μg/ml and 10 μg/ml) significantly increased cytoplasmic and nuclear Nrf2 levels and increased nuclear: cytoplasmic Nrf2 ratios (Figures 3C,D). We further investigated the effects of UVA exposure on Nrf2-mediated downstream gene expressions including GST and NQO-1 mRNA expression. Nrf2-target genes including GST and NQO-1 were reduced significantly in the UVA-irradiated cells (Figures 3E,F). Conversely, the treatment with HRF and HSP significantly prevented this reduction. In addition, these protective effects were also observed with the positive control, t-BHQ (0.2 μg/ml), an effective activator of Nrf2 and its target genes, GST and NQO-1 (Figures 3C–F).

### HRF and HSP Protected Against UVA-Induced Skin Damage in BALB/c Mice *in vivo*

**FIGURE 4:**
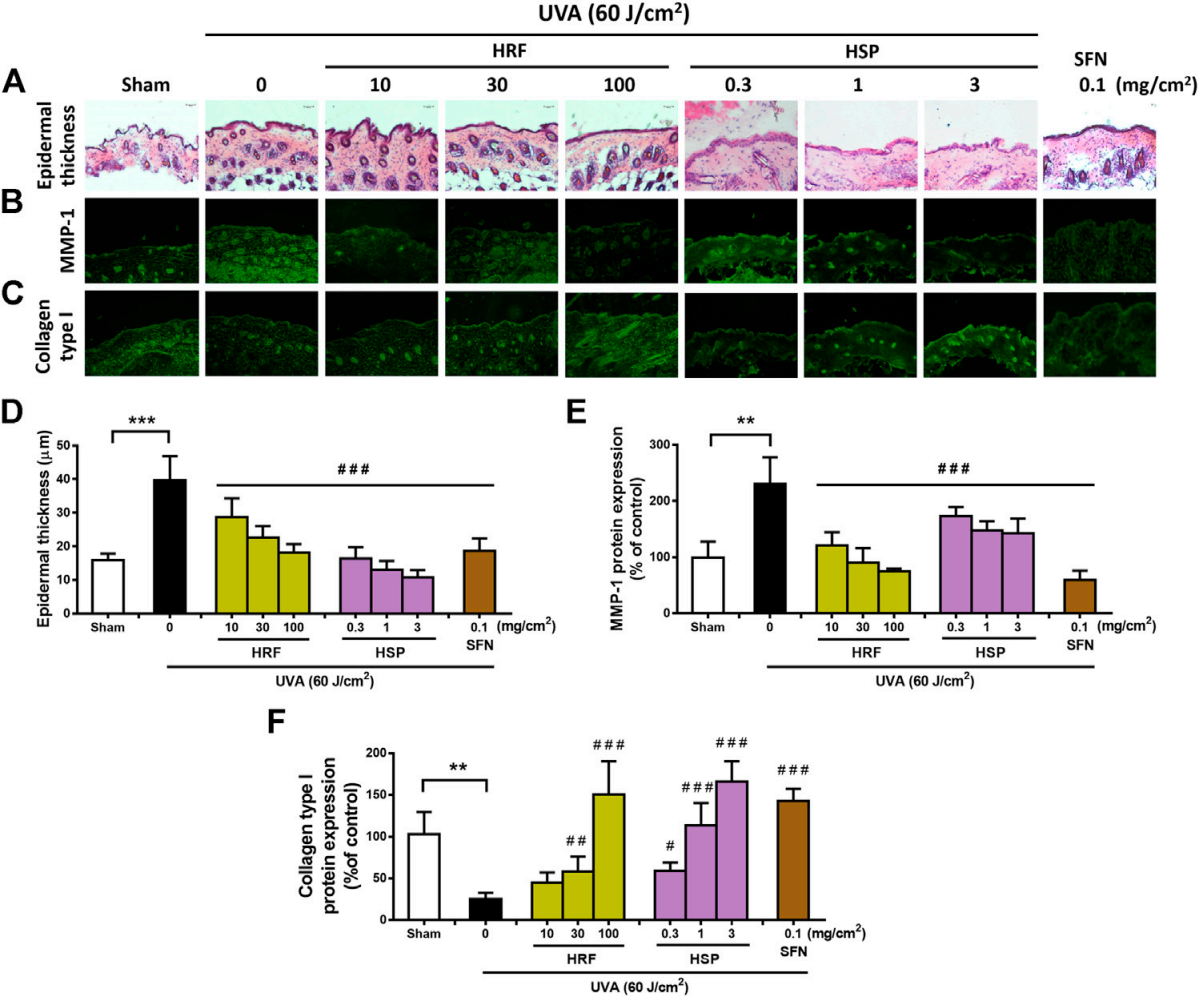
The protective effects of HRF and HSP on UVA-induced skin damage in the *in vivo* model. BALB/c mice were topically applied with 10, 30, and 100 mg/cm^2^ HRF and 0.3, 1, and 3 mg/cm^2^ HSP as well as the ethanol treatment and no compound treatment (0) on the dorsal skin for 1 h prior to each UVA irradiation (10 J/cm^2^/session 3 times a week for 2 weeks; a cumulative total dose of 60 J/cm^2^). Images of H&E staining for epidermal thickness **(A)** and the immunofluorescence of MMP-1 **(B)** and collagen **(C)** were collected at 24 h following the last UVA exposure. The summary graph with the statistical analysis of epidermal thickness **(D)** and the protein levels of MMP-1 **(E)** and collagen **(F)** was quantified by ImageJ and GraphPad Prism software. Data was shown as mean ± SD at the ×20 magnification (scale bar = 50 μm), *n* = 4. **p < 0.01, ***p < 0.001 vs. non-irradiated sham group by Student’s *t*-test. *#p* < 0.05; ##*p* < 0.01; ###*p* < 0.001 vs. the sham-irradiated group by one-way ANOVA Dunnett's test.

Our *in vitro* study implied that HRF and HSP showed the antioxidant and anti-aging properties in NHDFs irradiated with UVA. This study thus further examined the effects of the HRF and HSP on UVA-induced photoaging in a relevant *in vivo* mouse model. Since we previously optimized the timeline of *in vivo* treatment and the irradiation dose of UVA (Chaiprasongsuk et al., 2017), the HRF and HSP were topically applied to the mouse dorsal skin for 1 h prior to UVA irradiation (total cumulative dose 60 J/cm^2^). H&E and IF staining of skin tissue sections revealed that UVA treatment markedly induced epidermal thickness (Figures 4A,D) and MMP-1 protein expression (Figures 4B,E) as well as reduced collagen type 1 protein levels (Figures 4C,F). In contrast, the topical treatment of mouse skin with HRF (10–100 mg/cm^2^) and HPS (0.3–3 mg/cm^2^) led to the reduction of the epidermal thickness (Figures 4A,D) and MMP-1 protein expression (Figures 4B,E) as well as the induction of collagen type 1 protein levels (Figures 4C,F). As expected, positive control, sulforaphane (SFN) also provided these protective effects (Figures 4A–F).

### HRF and HSP Suppressed Downregulation of Nrf2 Levels and Its Downstream Signaling in UVA-Induced Photodamage of Mouse Skin *in vivo*

**FIGURE 5:**
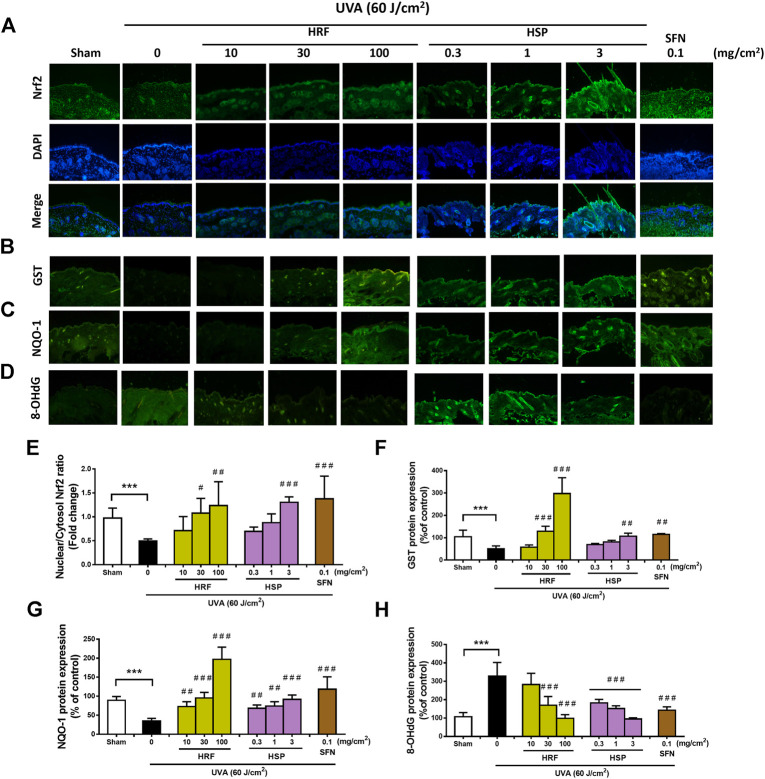
The modulation of the Nrf2-mediated antioxidant response by HRF and HSP in mouse dorsal skin irradiated with UVA. Dorsal skin was treated with compounds as previously describes and harvested at 1 h following the final irradiation. Images of immunofluorescence staining (FITC-conjugated secondary antibody staining indicated the location of Nrf2 (green by the anti-Nrf2 antibody. DAPI staining indicated the location of the nucleus (blue) and the merged image indicated the nuclear localization of Nrf2 **(A)**. The summary graph with the statistical analysis of the nuclear-to-cytosolic Nrf2 ratio **(E)** was quantified by ImageJ and GraphPad prism software and was expressed as mean ± SD, *n* = 4. The immunofluorescence of antioxidant proteins including GST **(B)**, NQO-1 **(C)**, and the oxidative DNA damage 8-OHdG **(D)**, were collected at 6 h following the final UVA exposure. The summary graph with the statistical analysis of the protein levels of GST **(F)**, NQO-1 **(G)**, and 8-OHdG **(H)** were quantified by ImageJ and GraphPad prism software and were expressed as mean ± SD, *n* = 4. ***p < 0.001 vs. non-irradiated sham group by Student’s *t*-test. *#p* < 0.05; ##*p* < 0.01; ###*p* < 0.001 vs. the sham-irradiated group by one-way ANOVA Dunnett's test.

Mechanistic studies have been previously reported that Nrf2 is required for protection against UVA-stimulated MMP-1 upregulation (Chaiprasongsuk et al., 2016). Our *in vitro* studies (Figure 3) supported the novel understanding of the inhibitory effects of the HRF and HSP on UVA-mediated reduced activity of Nrf2 in NHDFs, we thus further examined whether HRF and its potential bioactive compound, HSP, could protect against the skin photoaging through this pathway in mouse skin *in vivo*. At 1 h post-irradiation, the nuclear Nrf2 levels were significantly reduced in the mouse skin exposed to the UVA irradiation (60 J/cm^2^) (Figure 5A) and this reduction was dose-dependently inhibited by HRF and HSP (30–100 mg/cm^2^ and 3 mg/cm^2^) (Figure 5E). The HRF (30–100 mg/cm^2^) and HSP (3 mg/cm^2^) also markedly abrogated UVA-induced reduction of GST protein levels (Figures 5B,F). The decline of NQO-1 protein levels was also significantly inhibited by HRF and HSP in a concentration-dependent manner (10–100 mg/cm^2^ and 0.3–3 mg/cm^2^) in mouse skin exposed to UVA (Figures 5C,G). To evaluate the photoprotective effect of HRF and HSP on UVA-mediated oxidative DNA damage, the formation of 8-hydroxy-2′-deoxyguanosine (8-OHdG), a sensitive marker of oxidative DNA damage, was quantified in the mouse dermis at 1 h following the final UVA exposure with cumulative doses of 60 J/cm^2^. The results indicated that UVA irradiation increased the skin levels of 8-OHdG indicative of inducing oxidative DNA damage. However, HRF (30–100 mg/cm^2^) and HSP (0.3–3 mg/cm^2^) dose-dependently protected against UVA-induced oxidative DNA damage in the mouse skin (Figures 5D,H).

## Discussion

The development of botanical products including herbal formula as potential anti-photoaging products has gained attention in phytomedicine and dermatology research. Several plant extracts and their active ingredients have been investigated for their anti-photoaging effects against UVR-induced various MMPs in association with the antioxidative and anti-inflammatory actions in cultured keratinocytes and dermal fibroblasts as well as mouse skin (Binic et al., 2013). We and other studies observed that ROS generated during UVA exposure of the skin causes upregulation of MMPs, in particular MMP-1, responsible for the degradation of the ECM proteins including collagen that contributes to photoaging (Pittayapruek et al., 2016). We previously reported that the Thai Herbal HRF extracts protected against UVA-induced MMP-1 in keratinocyte HaCaT cells (Pluemsamran et al., 2013) and melanogenesis in B16F10 melanoma cells through activating Nrf2-dependent antioxidant defenses (Onkoksoong et al., 2018). In this study, we determined the anti-photoaging effects of HRF extract and its possible active ingredient, hesperetin (HSP), on normal human dermal fibroblasts *in vitro* and on mouse skin *in vivo* exposed to UVA in association with modulation of Nrf2-related antioxidant signaling.

Our previous studies observed the abilities of the HRF extracts to suppress UVA-induced MMP-1 activity in keratinocyte HaCaT cells *via* the promotion of antioxidant defense capacity. To support further development of the HRF extracts as potential photoprotective products, this is the first time the protective effects of HRF extracts on the skin were tested using *in vitro* and *in vivo* model of UVA-induced photoaging in association with modulation of Nrf2-mediated redox balance. Indeed, both HRF extracts and HSP, its possible bioactive constituent, decreased UVA-induced cytotoxicity, oxidative stress, MMP-1 induction, and collagen degradation in a concentration-dependent manner, in association with concomitant rises in Nrf2 nuclear translocation and its target gene levels in a concentration-dependent manner. These findings were also reproduced *in vivo* and both HRF extracts and HSP dose-dependently protected against UVA-mediated MMP-1 induction, collagen loss, and oxidative DNA damage. The HRF extracts and HSP also enhanced a substantial Nrf2 activation in mouse skin, together with the upregulation of antioxidant defense enzymes GST and NQO-1. We and other studies have reported that activation of Nrf2 is involved in the mechanisms underlying the photoprotective effect of phytochemicals and botanical extracts against UVR-induced damage of the skin cells including melanocytes and keratinocytes (Chaiprasongsuk et al., 2016; Chaiprasongsuk et al., 2017; Onkoksoong et al., 2018). Our findings strongly suggested that the photoprotective actions of HRF extracts and HSP at concentrations of 30 μg/ml and 10 μg/ml, protected against UVA-induced photoaging of NHDFs. As reported by our previous studies and others, the HRF or Benchalokawichian was demonstrated to exert the antipyretic and anti-inflammatory actions *in vivo* (Jongchanapong et al., 2018) as well as anti-allergic, anti-inflammatory antioxidant, anti-collagenase, and depigmenting actions *in vitro* (Pluemsamran et al., 2013; Onkoksoong et al., 2018; Juckmeta et al., 2019; Noysang et al., 2019). Furthermore, HSP, a citrus flavonoid, has previously shown the anti-inflammatory responses and antioxidant properties by inducing the Nrf2-antioxidant defense system including the GCL and NQO-1 detoxifying antioxidant signaling as well as providing the protective effects against oxidative damage and carcinogenesis (Parhiz et al., 2015; Roohbakhsh et al., 2015; Ren et al., 2016; Biesemann et al., 2018; Ferreira de Oliveira et al., 2020). *In vitro* and *in vivo* studies have previously suggested that Asian medicinal herbs containing HSP as a possible active ingredient were demonstrated to provide a therapeutic effect in the skin carcinoma treatment and play a role in anti-inflammation possibly mediated by Nrf2 signaling (Smina et al., 2015; Muhammad et al., 2019). Additionally, the distribution of HSP at a relatively high concentration was found in mouse skin following its oral intake (Takumi et al., 2011), and HSP accumulated in the skin could thus potentially reach the target site and achieve the pharmacological action (Dekker et al., 2005). Since the medicinal herbs have traditionally been used to treat skin problems, the topical treatment targeting Nrf2 was demonstrated to protect human skin against UV-induced erythema employed as a surrogate marker for cutaneous damage and skin cancer risk (Talalay et al., 2007). We and others have demonstrated that UVA exposure to skin cells induces a robust induction of oxidative stress involved in activation of MMP-1 signaling responsible for collagen degradation. Therefore, the anti-photoaging effects of HRF extracts and HSP might be due to activation of Nrf2 and its downstream antioxidant enzymes GST and NQO-1, resulting in the suppression of ROS generation and subsequently inhibited MMP-1 activation and collagen degradation. ROS generation in response to UVA irradiation was demonstrated to mediate the upstream kinases involved in activating protein-1 (AP-1) activation, leading to the upregulation of the MMP-1 gene (Chaiprasongsuk et al., 2017; Hseu et al., 2020). Thus, the mechanisms by which HRF extracts and HSP mitigated MMP-1 activity may involve their abilities to reduce ROS level, subsequently leading to downregulation of MAPK/AP-1/MMP-1 axis. Moreover, ROS was suggested to play a role in photoaging *via* stimulating pro-inflammatory mediators (e.g., TNF-α, IL-6, IL-1β)-mediated signaling cascades including MAPK-AP-1/NF-κB, leading to transcriptional regulation of MMP-1 (Pittayapruek et al., 2016; Subedi et al., 2017). HSP was also reported to possess anti-inflammatory effects via downregulation of pro-inflammatory cytokines (including TNF-α, IL-6, and IL-1β) and NF-κB signaling concomitant with activation of Nrf2 in various *in vitro* and *in vivo* models (Parhiz et al., 2015; Li and Schluesener, 2017; Lin et al., 2020). Therefore, anti-photoaging effects and mechanisms of HRF containing HSP could also involve the anti-inflammatory activity against UVA-induced MMP-1 upregulation in dermal fibroblasts.

Our observations revealed that the HRF extracts and HSP did not directly modulate Nrf2 activity nor photoaging parameters (MMP-1 and collagen) because treatment with the HRF and HSP alone for 30 min did not significantly promote Nrf2 nuclear translocation nor affect MMP-1 expression in non-irradiated mouse skin (Supplementary Figure S3). Therefore, as regards to abilities of the HRF extracts and HSP to mitigate UVA-induced ROS formation, the anti-photoaging effects of HRF extracts and its bioactive compound HSP might be attributed to indirect regulation of Nrf2 signaling and its activation is redox-dependent. Moreover, various phytochemicals present in herbs can provide photoprotective effects due to the sunscreen actions (Korac and Khambholja, 2011) and the phenolics with UVA absorption properties were previously observed in our study to indirectly promote Nrf2 activity (Chaiprasongsuk et al., 2016). Further study is thus needed to explore the photoprotective effects of the HRF consisting of complex mixtures of phytochemicals that might involve not only antioxidant but also sunscreen actions responsible for the HRF’s abilities to block UVA-induced ROS formation. The pharmacological effects of herbal medicines are attributed to the presence of different phytoconstituents, and compatible herbals formulated in polyherbal formulations could potentiate their effects. In addition to HSP suggested as a potential bioactive compound of the HRF, other phytochemicals such as hispidulin and gallic acid identified in the HRF extracts and its 5 components could also be responsible for the biological actions of the HRF. Thus, qualitative and quantitative analysis of the HRF’s bioactive constituents remain challenging due to their complexity and need further investigations.

Attempts have been made to develop Nrf2 activators as chemopreventive strategies including photoprotective agents for the prevention of photocarcinogenesis, although it should be taken into consideration that Nrf2 can be the double-edged sword in human cells and a harmful aspect of Nrf2 in the biology of skin cancer has been discussed (Kim et al., 2010; Sporn and Liby, 2012). Overexpression of Nrf2 and its downstream genes is also suggested to play a role in promoting cancer cell survival, growth and chemoresistance (Lau et al., 2008; Hayes and Dinkova-Kostova, 2017; Jessen et al., 2020). Hence, further clarification is warranted regarding the dual roles of Nrf2 in the biology of skin cancer.

## Conclusion

In summary, we have demonstrated that HRF extracts and their potential bioactive compound HSP prevented UVA-induced photoaging of NHDFs *in vitro* and mouse skin *in vivo via* suppressing oxidative damage and downregulating MMP-1-mediated collagen degradation through the regulation of cytoprotective Nrf2 activity. In this respect, the topical use of the HRF, a polyherbal formulation, may represent a complementary and alternative medicine strategy for preventing and/or delaying the skin photoaging through the restoration of redox homeostasis.

## Data Availability

The raw data supporting the conclusion of this article will be made available by the authors, without undue reservation, to any qualified researcher.
