# Supplementary material for: The Protective Effect of Polyherbal Formulation, Harak Formula, on UVA-Induced Photoaging of Human Dermal Fibroblasts and Mouse Skin via Promoting Nrf2-Regulated Antioxidant Defense
Source: Front Pharmacol. 2021 Apr 12;12:649820. doi: 10.3389/fphar.2021.649820 (PMC8072377; doi:10.3389/fphar.2021.649820)
Supplement: Supplementary file 1 [file datasheet1.docx]

Supplementary Material

The protective effect of polyherbal formulation, Harak formula, on UVA-induced photoaging of human dermal fibroblasts and mouse skin via promoting Nrf2-regulated antioxidant defense

**Jinapath Lohakul^1^, Anyamanee Chaiprasongsuk^2^, Saowanee Jeayeng^1^, Malinee Saelim^1^, Phetthinee Muanjumpon^1^, Saowalak Thanachaiphiwat^1^, Pinpat Tripatara^1^, Kittipong Soontrapa^1^, Natchagorn Lumlerdkij^3^, Pravit Akarasereenont^1,3^, Uraiwan Panich^1^***

^1^Department of Pharmacology, Faculty of Medicine Siriraj Hospital, Mahidol University, Bangkok 10700, Thailand. (J.L., S.J., M.S., P.M., S.T., P.T., K.S., P.A., U.P.)

^2^ Faculty of Medicine and Public Health, HRH Princess Chulabhorn College of Medicine Science, Chulabhorn Royal Academy, Bangkok, Thailand. (A.C.)

^3^Center of Applied Thai Traditional Medicine, Faculty of Medicine Siriraj Hospital, Mahidol University, Bangkok, 10700, Thailand. (N.L., P.A.)

*** Correspondence:**Uraiwan Panich, M.D., Ph.D.

Department of Pharmacology, Faculty of Medicine Siriraj Hospital, Mahidol University

2 Wanglang Rd., Bangkok Noi, Bangkok 10700, Thailand.

Phone: +66 818155925, Fax: +66 24197565, E-mail: uraiwan.pan@mahidol.ac.th

**
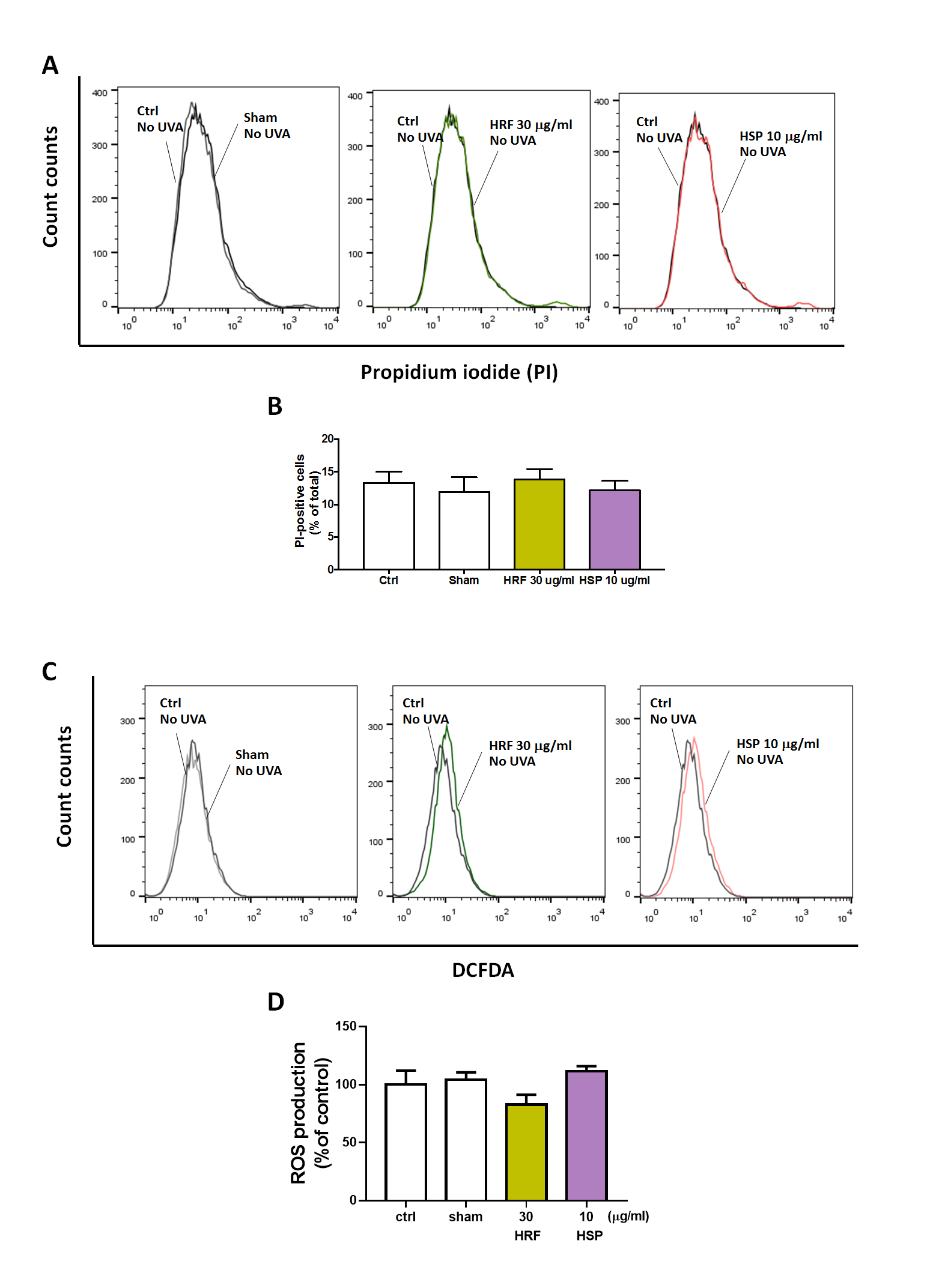
Supplementary Figures**

**Supplementary Figure 1.** **The reaction of HRF and HSP at the highest concentration involving in cytotoxicity and cellular oxidant generation in treated cells without UVA irradiation.**

The HRF and HSP treated in NHDF cells for 25 minutes were harvested to perform the flow cytometry of fluorescent staining, PI dye (% PI positive cells) and determine cytotoxicity. The representative flow histogram (A), and the summary graph analysis of the ethanol treated cells, HRF and HSP at the highest dose (30 µg/ml and 10 µg/ml, respectively) compared to unirradiated control (ctrl) cells (B). The flow cytometry of fluorescent staining, DCFDA dye and determined the ROS production (% of control). The representative flow histogram (C), and the summary graph analysis of the ethanol treated cells, HRF and HSP at the highest dose (30 µg/ml and 10 µg/ml, respectively) compared to unirradiated control (ctrl) cells (B) were expressed as mean ± SD, n = 4.

**
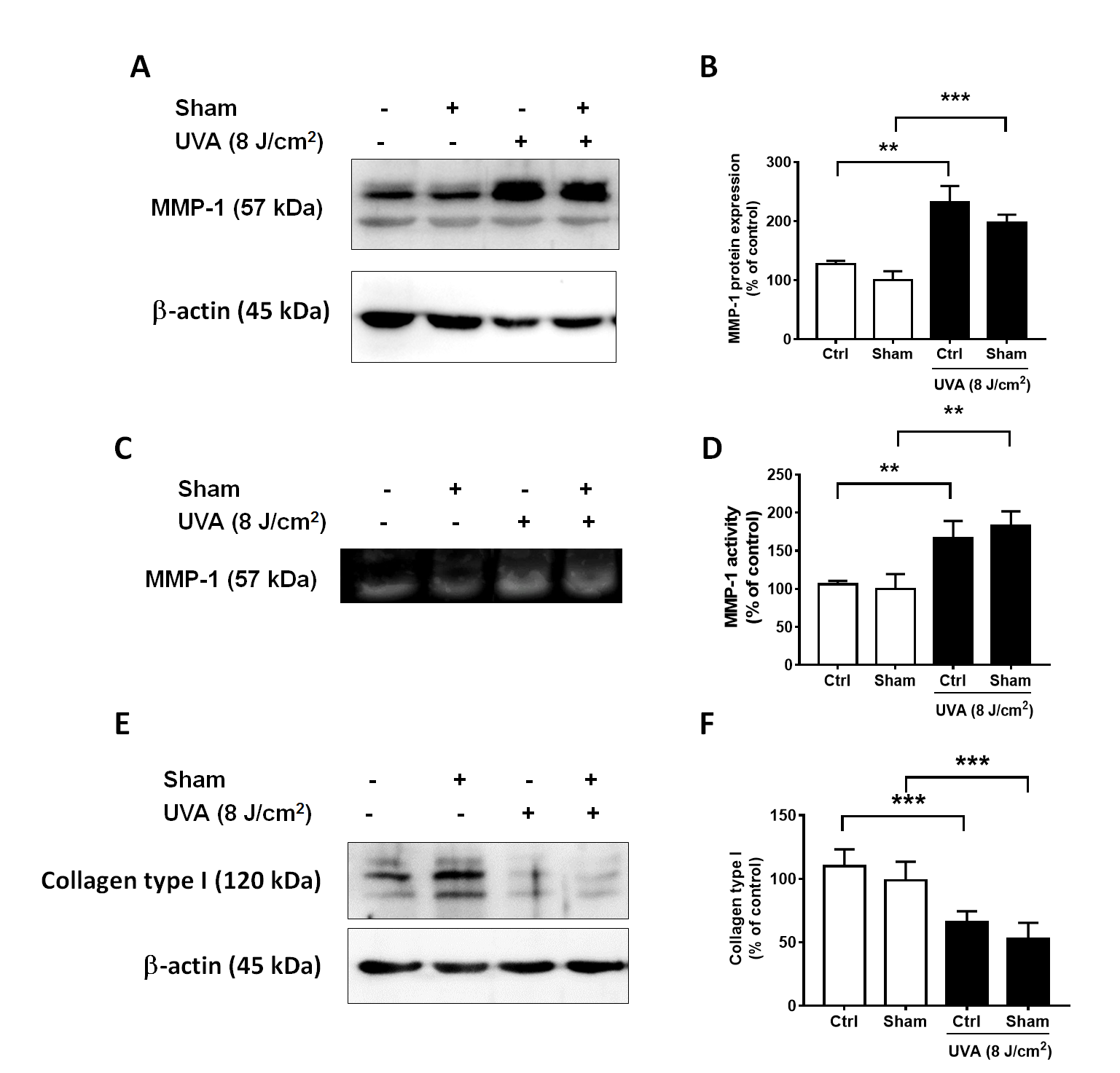
**

**Supplementary Figure 2.** **The effects of ethanol sham treated cells in MMP-1 protein expression and MMP-1 enzyme activity and collagen type I protein expression.**

The ethanol sham treated in NHDFs for 25 minutes prior to UVA (8 J/cm^2^) were harvested to perform the western blot analysis of MMP-1 protein expression, zymogram of MMP-1 enzyme activity and collagen type 1 protein expression. The representative blot pictures of MMP-1 protein expression (A), and the summary graph analysis of ethanol treated cells compared to UVA irradiated cells (B). The representative collagen zymography of MMP-1 enzyme activity (C), and the summary graph analysis of ethanol treated cells compared to UVA irradiated cells (D) and the western blot pictures of collagen type 1 protein expression (E), and the summary graph analysis of ethanol treated cells compared to UVA irradiated cells (F) were quantified by ImageJ and GraphPad prism software and were expressed as mean ± SD, n = 4. ***P <* 0.01, ****P* < 0.001 versus non-irradiated sham group by Student’s t-test.


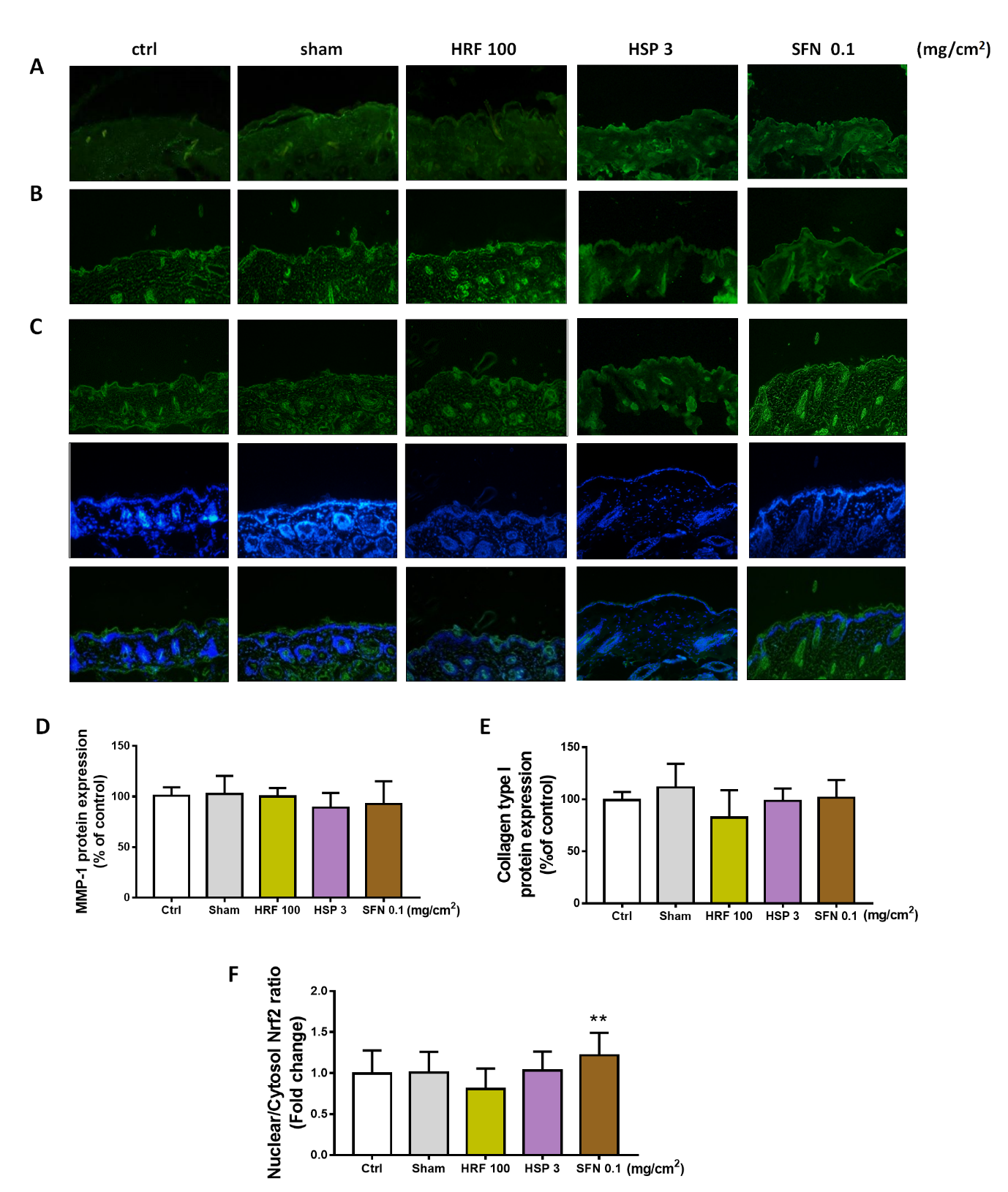


**Supplementary Figure 3.** **The effects of HRF and HSP at the highest concentration involving in MMP-1, collagen type I protein expression and nuclear-to-cytosol Nrf2 ratio without UVA irradiation.**

BALB/c mice were topically applied with HRF 100 mg/cm^2^ and HSP 3 mg/cm^2^ as well as the ethanol treatment and no compound treatment (ctrl) on the dorsal skin for 1 h (3 times a week for 2 weeks). The immunofluorescence analysis of MMP-1 (A) and collagen (B) were performed at 24 h following the last UV treatment session. Images of IF staining (FITC)-conjugated secondary antibody indicated the location of Nrf2 (green) by the anti-Nrf2 antibody. DAPI staining indicated the location of the nucleus (blue) and the merged image indicated the nuclear localization of Nrf2 (C). The summary graph with the protein levels of MMP-1 (D) and collagen (E) and nuclear-to-cytosolic Nrf2 ratio (F) were quantified by ImageJ and GraphPad Prism software. Data were shown as mean ± SD, n = 4.
